# Advanced Microscopy Techniques for Molecular Biophysics

**DOI:** 10.3390/ijms24129973

**Published:** 2023-06-09

**Authors:** Laura Barsanti, Lorenzo Birindelli, Francesca Sbrana, Giovanni Lombardi, Paolo Gualtieri

**Affiliations:** 1 Istituto di Biofisica, CNR, Via Moruzzi 1, 56124 Pisa, Italy; laura.barsanti@ibf.cnr.it (L.B.); lorenzo.birindelli@ibf.cnr.it (L.B.); 2 Istituto di Biofisica, CNR, Viale Marini 6, 16149 Genova, Italy; francesca.sbrana@ibf.cnr.it; 3 Istituto di Scienza e Tecnologia dell’Informazione, CNR, Via Moruzzi 1, 56124 Pisa, Italy; giovanni.lombardi@isti.cnr.it

**Keywords:** microspectrophotometry, super-resolution, stimulated emission depletion microscopy (STED), holotomography, *Euglena gracilis*, kleptoplastids, trout photoreceptors

## Abstract

Though microscopy is most often intended as a technique for providing qualitative assessment of cellular and subcellular properties, when coupled with other instruments such as wavelength selectors, lasers, photoelectric devices and computers, it can perform a wide variety of quantitative measurements, which are demanding in establishing relationships between the properties and structures of biological material in all their spatial and temporal complexities. These combinations of instruments are a powerful approach to improve non-destructive investigations of cellular and subcellular properties (both physical and chemical) at a macromolecular scale resolution. Since many subcellular compartments in living cells are characterized by structurally organized molecules, this review deals with three advanced microscopy techniques well-suited for these kind of investigations, i.e., microspectrophotometry (MSP), super-resolution localization microscopy (SRLM) and holotomographic microscopy (HTM). These techniques can achieve an insight view into the role intracellular molecular organizations such as photoreceptive and photosynthetic structures and lipid bodies play in many cellular processes as well as their biophysical properties. Microspectrophotometry uses a set-up based on the combination of a wide-field microscope and a polychromator, which allows the measurement of spectroscopic features such as absorption spectra. Super resolution localization microscopy combines dedicated optics and sophisticated software algorithms to overcome the diffraction limit of light and allow the visualization of subcellular structures and dynamics in greater detail with respect to conventional optical microscopy. Holotomographic microscopy combines holography and tomography techniques into a single microscopy set-up, and allows 3D reconstruction by means of the phase separation of biomolecule condensates. This review is organized in sections, which for each technique describe some general aspects, a peculiar theoretical aspect, a specific experimental configuration and examples of applications (fish and algae photoreceptors, single labeled proteins and endocellular aggregates of lipids).

## 1. Introduction

Many digital microscopy techniques have been developed in the life sciences to study organisms and the complex molecular processes occurring within them. Among the many digital microscopy set-ups available now is confocal laser scanning microscopy (CLSM) [1,2,3], which increases the axial and lateral optical resolution of the formed images, blocking out-of-focus light by means of spatial pinholes, while fluorescence correlation spectroscopy (FCS) [4] uses correlation analysis to determine changes in fluorescence intensity. Other set-up examples are: fluorescence resonance energy transfer microscopy (FRET) [5,6], which measures the non-radiative energy transfer of labeled proteins to investigate their interactions; structured illumination microscopy (SIM) [7], which achieves enhanced resolution at low light intensity using a patterned illumination technique; STORM (stochastic optical reconstruction microscopy) [8] and PALM (photo activated localization microscopy) [9], which separates individual proteins using photo-switchable or activable fluorochromes.

This review will cover the theory and applications of microspectrophotometry (MSP), super-resolution localization microscopy (SRLM) with some of its different approaches and holotomographic microscopy (HTM). These three analytical techniques are well-suited to perform intra-vital measuring with high spatial resolutions of the volumetric architecture of organelles and molecules and their interactions. MSP measures the absorption spectra of photoreceptive structures and photosynthetic structures in the visible range. SRLM localizes single fluorescent proteins in the cytoplasm with high precision and determines their true size. HTM, which combines holographic and tomographic techniques in a single instrument, measures quantitatively the phase of lipid and protein condensates and allows their separation and 3D reconstruction. The organization of this review in sections provides a first general description of each technique, an analysis of a distinctive aspect of the theory on which the technique is based and the description of a specific experimental configuration, together with an example of an experiment conducted with that technique (fish and algae photoreceptors, single labeled proteins and endocellular aggregates of lipids).

## 2. Microspectrophotometry

### 2.1. General Description

MSP has been used for the identification of the chromophores present in photoreceptive structures, providing information about possible mechanisms of energy transfer. Using absorption MSP, the integrity of the subcellular component is not disturbed, and it is possible to examine its intact physiological functions in the uninjured cell. MSP can achieve quantitative measurements of absorbance even lower than 10^−2^ in a subcellular compartment whose dimensions can be as low as 0.5 μm [10]. Because of the fundamental key connections existing between optical parameters and properties of molecular structures, MSP allows assessments of state changes of the molecules present in the sample (e.g., photoreceptive protein photocycle, chlorophyll degradation to pheophytin [11,12,13,14,15], or their aggregation degree). In many cases the liability and reversibility of these changes make MSP the only possible method of investigation. In the case of cellular compartments containing different pigments as the chloroplasts (chlorophylls, carotenoids and phycobiliproteins), the relative contribution of the individual pigments to the absorption spectra is often difficult to identify and quantify, since the ability to discriminate among the different contributions depends upon the robustness of the chosen technique. The curve-fitting routine, which relies upon templates of the absorption spectra of pigments previously measured in the natural environment, has proved to be an efficient and reliable method, as shown by Coltelli et al. (2016) [16].

MSP allows also fluorescence measurements. This method has been applied to many biologically active fluorochromes, both endogenous and exogenous [17,18]. In case of fluorescence determination, the background is much reduced and therefore fluorescence spectroscopy of living cell compartments is more sensitive compared to absorption spectroscopy. For all practical purposes the sensitivity of detection in the case of fluorescence microscopy is not limited by the signal-to-noise ratio, but rather by the presence, virtually unavoidable, of fluorescent contaminants. For a comprehensive description of fluorescence and its quantitative applications and limitations refer to chapter 7 “Working with light” of the book of Gualtieri and Barsanti, 2023 [19].

### 2.2. Theory

In absorption MSP the factors that determine for each measuring wavelength the radiant flux I, expressed in W, impinging on the photoelectric device (photomultiplier, CCD camera) are:

(a) SR, the spectral radiance of the lamp, expressed in W cm^−2^ sr^−1^ nm^−1^, i.e., the radiant flux per unit area, solid angle and spectral bandwidth;
(b) SB, the spectral bandwidth, expressed in nm, for each measuring wavelength;
(c) OF, the optical flux of the optical system, i.e., the ability of the optical system to transfer light energy. OF is expressed in cm² sr and is a purely geometric quantity applied to the volume through which the light is transferred. In wide-field microscopy (WFM) the OF is about 10^−3^ cm^2^ sr, and the smallest optical flux giving a reliable measurement value is about 10^−8^ cm^2^ sr.

The OF depends on F, which is the area of either the aperture diaphragm or diaphragm image, and the effective numerical aperture of the microscope system NA as in the following formula

OF = πF NA^2^ (1)

(d) TR, the total transmittance of the optical system, is a measure of the percentage of light that remains after loss by absorption, reflection and diffraction of optical components (i.e., lens, mirrors, ...);
(e) ILS, the interactions of the light with the sample, is related to the absorption cross section of absorbing molecules and their number, and it is a measure of the percentage of light that remains after the sample absorption.

MSP measurements are based on a comparison of the two radiant fluxes I_i_ (incident flux) and I_t_ (transmitted flux). I_i_ results from the interaction of light with the background, and I_t_ results from the interaction of light with the sample. Therefore, the fundamental laws of MSP can be set out as follows:

I_i_ = SR SB OF TR (2)

I_t_ = SR SB OF TR ILS (3)

and the absorbance of a sample A_s_ is calculated as:

A_s_ = log(I_i_) − log(I_t_) = −log(ILS) (4)

For the light intensities used in MSP, the relative contribution of dark current noise, i.e., the irreducible intrinsic limit of any photoelectric measurement, is larger than that of other possible noise sources. For example, the mean-square photoelectron noise or shot noise current (I_n_) at the level of the cathode of a photomultiplier is given by

I^2^_n_ = e^2^kI_i_R/τ (5)

where e is the charge of the electron, k the quantum efficiency (the ratio between generated photoelectrons and incident photons at the cathode surface), I_i_ the incident radiant flux at the object plane, R the area of object illuminated and τ the detection time.

The mean-square shot noise involved in a measurement is contributed essentially by the incident beam, and the average photoelectric signal current difference (I_s_) is given by

I_s_ = I_i_ − I_t_ = ekFI_i_R (6)

where F is the fraction of the incident light absorbed by the sample.

The signal-to-noise ratio (SNR) of a shot-noise-limited photometer using the determined values of both signal and noise is:

SNR = I_s_/I_n_ = F(kI_i_τR)^1/2^ (7)

Since the detectivity is quantitatively proportional to the signal-to-noise ratio, the chances of detecting a substance that absorbs a fraction of the incident light (F) are improved by selecting a detector with a high quantum efficiency, k; by using a high flux density, I_i_, and a long exposure time τ; and by collecting light from a larger sample (higher R) [2].

### 2.3. A Working Set-Up

Different configurations exist for absorption MSP. The reviews by Gualtieri (1991) [2], Evangelista et al., (2006) [20] and Barsanti et al., (2007) [21] give well-described examples of these set-ups.

The MSP configuration used for the experimental examples reported in this review is characterized by a high-quality inspection probe, consisting of a bundle of 20 light-guides inserted in the back focal plane (entrance pupil) of a port of the binocular tube of a microscope. Each light-guide acquires the light transmitted by the region of interest (ROI) in the sample. The inspection probe is vertically aligned at the level of the exit pupil and focused onto a flat field imaging concave grating polychromator, which acts as a diffraction grating to disperse the impinging light into separate wavelengths. The dispersed image of the probe is in turn focused and imaged onto the target of a digital slow-scan cooled CCD camera, with a high signal-to-noise ratio.

**Figure 1:**
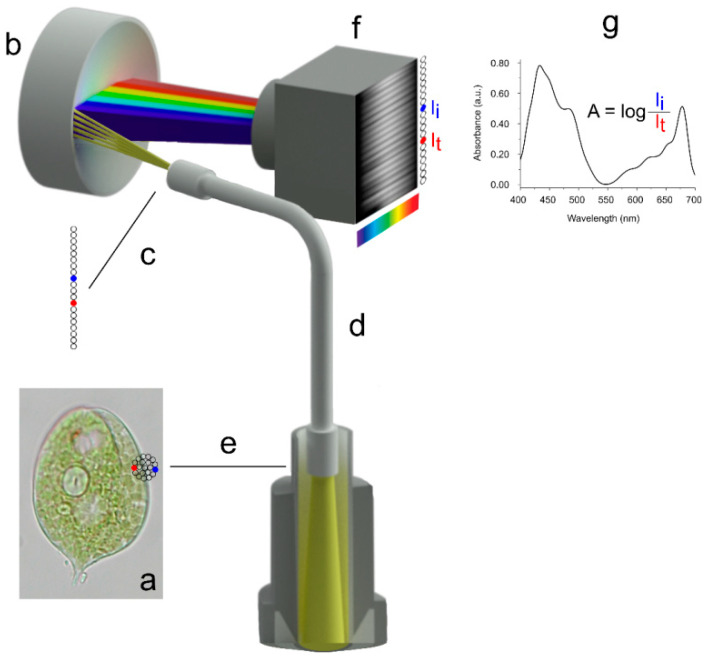
Schematic drawing of a part of the hardware platform used for microspectrophotometry: (**a**) the sample: a *Phacus* sp. cell (30 μm); (**b**) polycromator; (**c**) exit pupil of the optical fiber showing the 20 aligned light-guides; (**d**) optical fiber; (**e**) entrance pupil of the optical fiber and the central bundle of 20 light-guides; (**f**) CCD camera, the target on the back of the camera shows the image dispersed by the polychromator on its target; and (**g**) absorption spectrum of chloroplast *Phacus*. Adapted with permission from Ref. [22]. Copyright 2007, Copyright Paolo Gualtieri.

For the measurement, the inspection probe is centered on the ROI in such a way that at least one or two light-guides are positioned on an empty field, while the others are positioned on the absorbing compartment (Figure 1). The light-guides positioned on the compartment acquire the transmitted radiant flux (I_t_), while those positioned on the empty field acquire the incident radiant flux (I_i_). Absorbance measures in the visible range can be obtained in a compartment as small as 0.25 µm (cf. Equation (8)).

### 2.4. Experimental Example 1: MSP on Retinal and Extra-Retinal Photoreceptors of Teleost Fish

MSP has been used to clarify the correlations between cellular morphology and function with the pigment content of retinal and extra-retinal fish photoreceptors [23,24].

**Figure 2:**
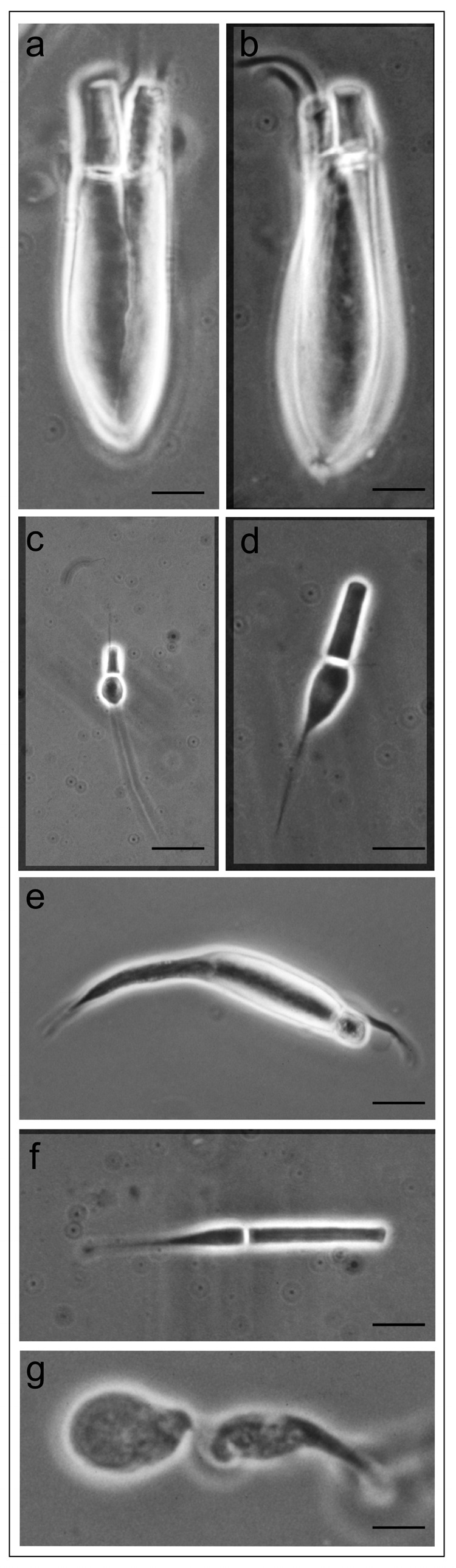
Phase contrast micrographs of retinal and extra-retinal photoreceptors of the trout *Salmo irideus*: (**a**) twin green cones; (**b**) unequal red/green cones; (**c**) uv single cone; (**d**) blue single cone; (**e**) green single cone; (**f**) rod; and (**g**) pineal photoreceptor. Scale bar 10 μm. Adapted with permission from Ref. [23]. Copyright 1993, Copyright Paolo Gualtieri.

**Figure 3:**
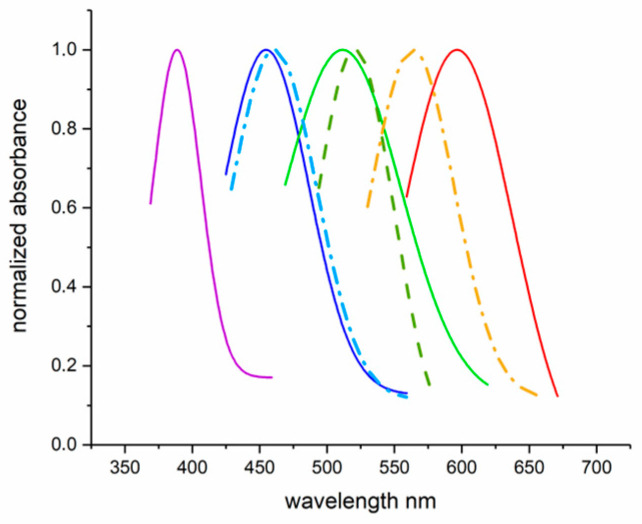
Absorption spectra measured by MSP of the different fish photoreceptors shown in Figure 2. Rod (green grass dashed), pineal (cyano and orange dot-dashed), and cones (purple, blue, green and red continuous lines). See text for details. Adapted with permission from Ref. [23]. Copyright 1993, Copyright Paolo Gualtieri.

The retinas of freshwater teleosts, such as *Salmo irideus*, possess rods, single cones and equal and unequal double cones. These fish also possess pineal photoreceptors, which have an extra-retinal location (Figure 2). MSP measurements identify the presence of different opsin proteins, also in the same photoreceptor, which absorb in the blue, green and red regions of the visible spectrum (Figure 3). MSP revealed also the presence of ultraviolet cones in the retina of juvenile trout, an advantage in shallow freshwater (less than 1 m) to detect planktonic food, which scatter ultraviolet wavelengths. Two distinct visual pigments were recorded in the pineal cells, demonstrating that opsin proteins are involved in the day–night rhythms of neuroendocrine activity (circadian clock) (Figure 3).

### 2.5. Experimental Example 2: Which Is the Prey?

This example has been selected to demonstrate the effectiveness of the spectral envelop decomposition developed by Coltelli et al., (2016) [16].

A number of dinoflagellate species lacking their own chloroplasts are known to retain functional chloroplasts (kleptoplastids) from ingested cryptophyte algal prey, which allow them to acquire photosynthetic capability and rely on its metabolic products [25]. Cryptophytes possess chlorophylls *a* and *c*_2_, phycobilins (phycoerythrin or phycocyanin) and alloxanthin, which is the carotenoid considered their taxonomic marker [19,26,27]. These pigments allow the algae to absorb a wide range of wavelengths and are optimized for the blue and green wavelengths penetrating deep in natural waters.

MSP was used to verify the nature of the pigment content of the predator dinoflagellate *Gymnodinium acidotum* and characterize the pigment present in three different possible cryptophyte preys. The comparison of the different spectroscopic patterns (difference spectra) allowed the identification of the cryptophyte preyed upon by the dinoflagellate [28].

**Figure 4:**
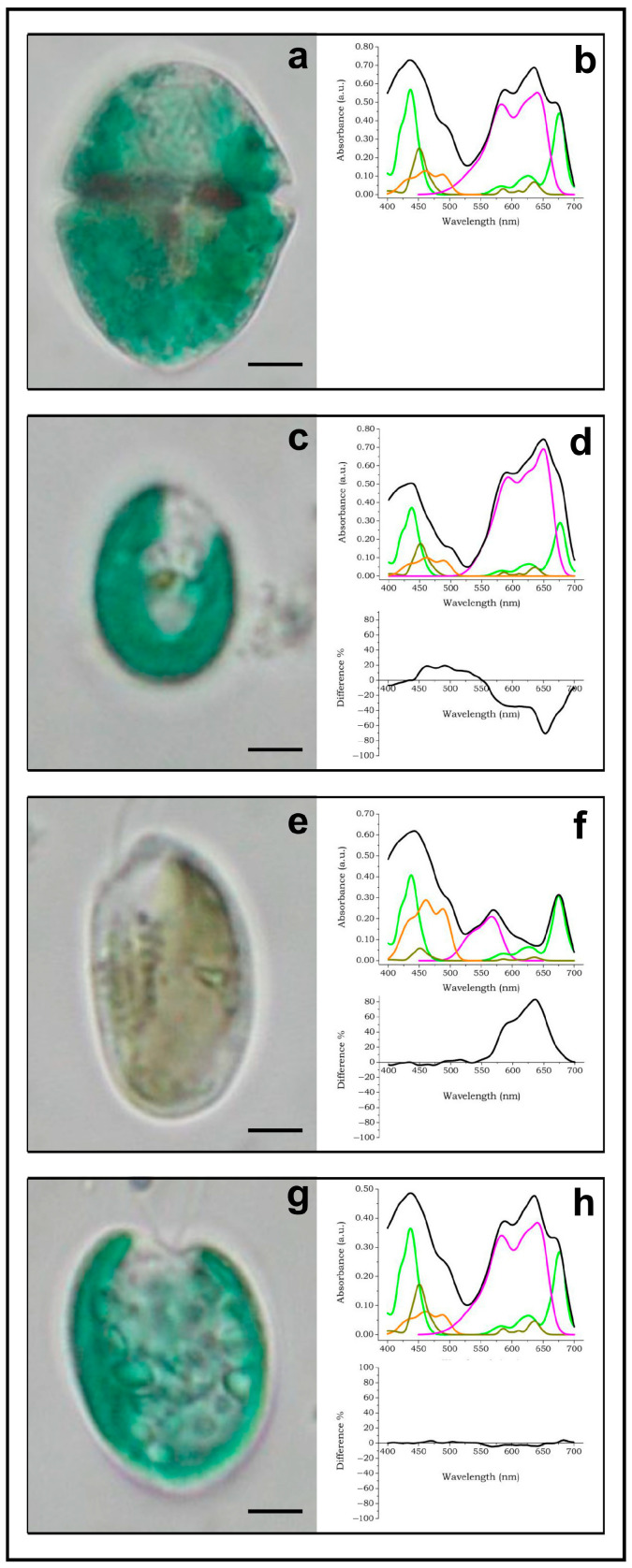
Absorption spectra of *Gymnodinium acidotum* and its possible preys. The identification of the prey was performed by MSP. Bright green line: chlorophyll *a*; dark green line: chlorophyll *c*_2_; orange line: carotenoids; purple lines: phycobilins; black line: spectral envelope. See text for details, scale bar 10 µm. Adapted with permission from Ref. [28]. Copyright 2009, Copyright Paolo Gualtieri.

The decomposed absorption spectrum of *Gymnodinium acidotum* highlights the presence of chl *a* (bright green line), chl *c*_2_ (dark green line), alloxanthin (orange line) and phycocyanin 645 (purple line) (Figure 4a,b).

The absorption spectrum of the first possible prey, an unknown blue–green cryptophyte, decomposes into chlorophyll *a*, chlorophyll *c*_2_ and alloxanthin (Figure 4c,d). Though the color of this cell is almost indistinguishable from that of the predator, the spectrum decomposition revealed that the absorption maximum is red-shifted at 650 nm and the absorbance in the region of phycobilins is much higher than the absorbance of chlorophylls. The difference spectrum (Figure 4d, bottom part) reveals that the dinoflagellate has a different spectral distribution with respect to the cryptophyte (χ^2^ = 74.88, *p* < 0.1), possessing more chlorophyll and less phycocyanin. Moreover, the negative peak at 650 nm confirms that the phycocyanin of the dinoflagellate is different from that of the cryptophyte and excludes the possibility that *Gymnodinium* preys upon this cryptophyte.

The absorption spectrum of *Cryptomonas* decomposes into chlorophyll *a*, chlorophyll *c*_2_, alloxanthin and phycoerythrin 566 (Figure 4e,f). The difference spectrum (Figure 4f, bottom part) reveals a significant positive area between 550 nm and 700 nm (χ^2^ = 466.5, *p* < 0.1), due to both the predator phycocyanin 645 and the *Cryptomonas* phycoerythrin 566. Also in this case, the spectral characteristics exclude the possibility that *Gymnodinium* preys upon *Cryptomonas*.

The absorption spectrum of *Chroomonas* decomposes into chl *a*, chlorophyll *c*_2_, alloxanthin and phycocyanin (Figure 4g,h). The difference spectrum (Figure 4h, bottom part) reveals an almost perfect match of the two spectra (χ^2^ = 0.77, *p* < 0.01), indicating that the pigmentation of *Gymnodinium* is due to the engulfment of *Chroomonas* cells.

## 3. Super-Resolution Localization Microscopy

### 3.1. General Description

SRLM has been used for imaging the molecular structures present in a cell with nanometric scale accuracy, providing information about their possible functioning [29]. Unlike the intracellular compartment described so far (photoreceptors and chloroplasts), which are characterized by endogenous chromophores, the structures resolved by SRLM (nuclear pores, chromatin complexes and cytoscheletal filaments) must be labeled by photo-switchable or activable fluorophores.

The lateral resolution_x,y_ limit of a conventional wide-field microscope is about 250 nm, while the axial resolution_z_ is about 450–700 nm. This limit is the fixed size of the spread of a single point of light that is diffracted through a microscope, which is defined as the point spread function (PSF). The limit is also a measure of the minimum size point source or object that can be resolved by a microscope. Objects smaller than the PSF appear to be the same size as the PSF in the microscope, and objects that are closer than the width of the PSF cannot be distinguished as separate.

A commonly used measure of the PSF width is the Rayleigh criterion R:

R = 0.61λ/NA(8)

where NA is the numerical aperture. Any microscopy technique that overcomes the resolution limit of conventional light microscopy by at least a factor of two is considered a super-resolution technique. Super-resolution techniques break the diffraction limit by temporally or spatially modulating the excitation light.

SRLM relies on molecular localization, in which a small subset of labeled fluorophores are stochastically activated with a probability of activation proportional to the intensity of the activation laser [30]. The centers of the individual excited fluorophores are then determined by localization algorithms with nanometer precision, and the final reconstructed image is obtained after accumulating localized fluorophore positions from tens of thousands of image frames. Therefore, SRLM is largely a computational imaging technique, built upon a simple configuration of a wide-field fluorescence microscope. The resolution of the reconstructed super-resolution image depends on the performance of localization accuracy algorithms and the density of fluorophores (hence the number of photons detected).

SRLM controls the fluorescence emission of fluorescent probes in time using either deterministic approaches as in stimulated emission depletion microscopy (STED) [31,32] and structured illumination microscopy (SIM) [7,33] or stochastic single-molecule localization approaches including photoactivated localization microscopy (PALM) [34], fluorescence photoactivation localization microscopy (FPALM) [35], stochastic optical reconstruction microscopy (STORM) [36] and direct STORM (dSTORM) [37,38]. One of the best results of SRLM was obtained by Loschberger (2012) [39], who measured the structure of a nuclear pore complex with a lateral resolution of about 15 nm using a dSTORM approach.

Table 1 compares the different SRLM techniques, highlighting their merits, limitations and main characteristics (resolutions_x,y,z_, simplicity, live imaging, time needed to build the image).

With super-resolution fluorescence microscopy, new challenges can be mastered and scientists are now capable of following the pathway of an individual molecule inside a living cell, observing proteins as they create synapses between nerve cells in the brain and resolving the aggregation of proteins involved in Parkinsons, Alzheimers and Huntington’s diseases [40]. In addition to biology, SRLM is useful also in other fields, such as materials science and nanotechnology. For example, SRLM is used to study the structure of materials at nanoscale, which is important for developing new technologies [41].

### 3.2. Theory

Super-resolution techniques, which belong to a more general problem of the extension of frequencies beyond those allowed by a linear system (e.g., WFM), are limited by noise [42,43]. In the case of a noise-free image, we will show to which theoretical limit the lateral resolving power of the microscope can be increased in a way compatible with Heisenberg’s uncertainty relation [44,45].

In an optical system, a single idealized point source emitter, located in an object plane x, forms a spatial radiance distribution G(y) in an image plane y. G(y) is the PSF of the optical system and represents the probability density function (PDF) of a photon position [46,47]. This is mathematically expressed as:

G(y) = (α/π) sinc^2^(αy) with α= 2 π a/λf (9)

where λ is the wavelength, 2a is the slit width and f the image conjugate focal distance.

The optical transfer function (OTF) in an optical system is a mathematical entity describing the response of an image system as a function of spatial frequency. It is given by the Fourier transform in the frequency domain ω of Equation (9) and is expressed as S(ω). If the noise-free spatial radiance distribution G(y) is continued over the whole image space, S(ω) takes the value 0 outside a determined frequency range. This is mathematically expressed as:

S(ω) = Tri(ω/2α)(10)

where Tri(x) is the triangular function, i.e., Tri(x) = 1 − |x| for |x| ≤ 1 and 0 elsewhere.

Using super resolution we can enrich the image with spatial frequencies that have been cut off by the microscope, achieving more accurate and precise details of the object.

Is this enrichment compatible with Heisenberg’s uncertainty relation? Following Frieden [48], we can express the first and second moment of G(y) as:

m_1_ = ∫ (α/π) sinc^2^(αy) y dy = 0 (11)

m_2_ = ∫ (α/π) sinc^2^(αy) y^2^ dy = ∞ (12)

The first moment m_1_ is equal to 0 since its mean value is at the peak of the curve G(y), i.e., the average position of a photon in the diffraction pattern can be considered its most likely single position. The second moment m_2_ is the measurement of the average spread; it states that a photon suffers infinite average spread in its diffraction pattern, i.e., the finite probability exists, however small, of finding the photon over the entire image plane. The value of m_2_ is equal to the opposite of the second derivative of the OTF, i.e., the value of the function S(ω) at the origin (x = 0):

m_2_ = −S″(0) = −(−∞) = ∞ (13)

The cusp at the origin of S(ω) accounts for the fact that m_2_ is equal to ∞ since it has a discontinuous slope. Heisenberg’s uncertainty relation [44] states that if a photon has an x-component of momentum μ, when it is in position x of the diffraction pattern, the spreads σ in x and μ obey:

σ_x_ σ_μ_ ≥ h/4π (14)

where h is Plank’s constant.

Therefore, the increase of the frequency range of the OTF obtained with a super-resolution procedure would always produce an OTF with a cusp in the origin and with an infinite σ_x_ in agreement with Heisenberg’s relation. Only the OTF without a cusp in the origin (i.e., a straight line), would not satisfy Heisenberg’s uncertainty relation. Practically speaking, the resolution improvement is strictly related to the signal-to-noise ratio of the system [1], and the localization accuracy ζ depends essentially on the photon noise (pixel noise and back ground noise are usually negligible):

ζ ≅ s/N^1/2^ (15)

where s is the standard deviation of the fitting localization function and N is the number of captured photons.

### 3.3. A Working Set-Up

**Figure 5:**
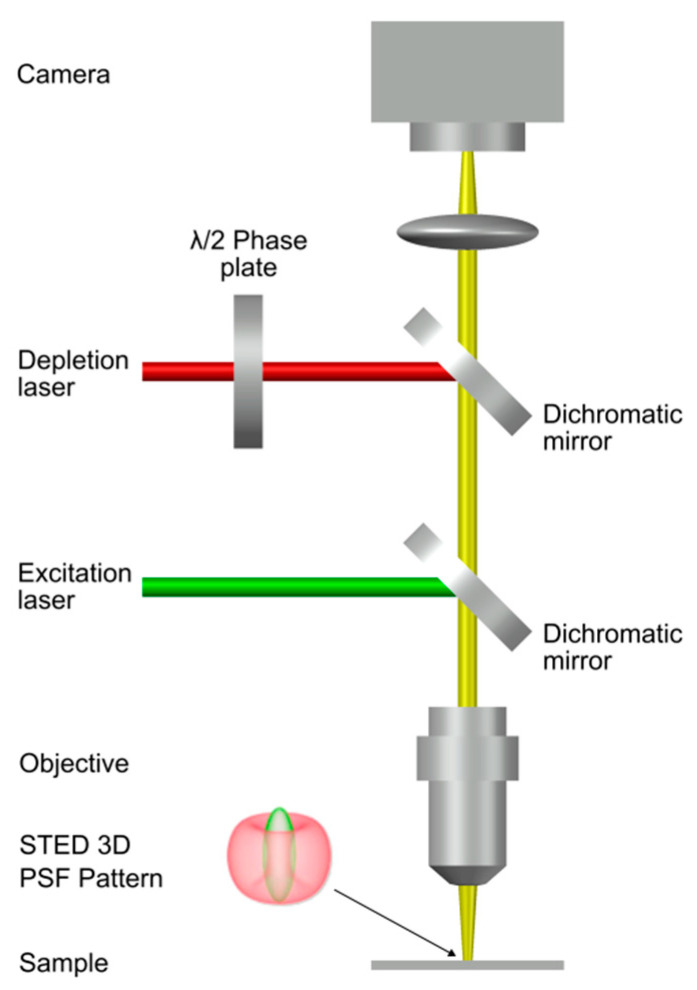
A typical STED optical train configuration. Adapted with permission from Ref. [29]. Copyright 2014, Copyright Christoph Braeuchle.

A very reliable and effective deterministic approach used in SRLM is stimulated emission depletion microscopy (STED) (Figure 5). The hardware set-up typically includes a confocal microscope equipped with a pair of synchronized pulsed lasers [29]. The first laser is a picosecond diode laser; it produces a diffraction limited scanning spot that excites the fluorescent proteins present in the area. The second laser is the STED laser, an intense red-shifted laser that quenches the emission of the proteins in a doughnut-shaped region centered in the focus of the excitation spot [49]. The emission of proteins localized in the center of the doughnut is not quenched and is detected by single-photon sensitive detectors (e.g., single photon avalanche diodes) [50]. By oversaturating the depletion, the fluorescence spot is reduced to dimensions below the diffraction limit thus producing a super-resolved image [29].

The rate of stimulated emission depletion (k_STED_) is given by

k_STED_ = φ I_STED_ (16)

where φ is the fluorophore cross-section and I_STED_ is the radiant flux of the STED laser.

Oversaturating the depletion requires a k_STED_ much larger than the fluorescence decay τ_Fl_ that is given by the inverse of the lifetime of the excite state S_1_, (about 1–5 ns). Therefore, to operate with a moderate average power, the excitation and the STED beams are implemented as synchronized pulse trains, whose duration is a fraction of τ_Fl_ (about 0.2 ns) [51,52].

The lateral resolution of the resulting spot is determined by I^max^_STED_/I_sat_, which is the saturation factor. This is the ratio between the maximal intensity in the STED depletion beam I^max^_STED_ and I_sat_, the intensity at which the probability of fluorescence emission is reduced by half and described by the following Equation:

Resolution ≅ λ/[2η sinα (1 + I^max^_STED_/I_sat_)^1/2^] (17)

In principle, since (ηsinα) is the NA of the doughnut, which is constant, the resolution can be arbitrarily increased by increasing I^max^_STED_ [53] (see the Section 3.2 Theory).

For example, at the typical 80 MHz repetition rate of mode-locked lasers (green dye Atto532), the average power and pulse energy is 1.5 mW and 0.3 nJ, respectively [54]. Larger I^max^_STED_ and hence much narrower focal spots have been reached only with a red dye [55].

### 3.4. Experimental Example 1: Single-Molecule Tracking and Imaging

The localization of proteins inside bacteria is possible because they are not confined into specific subcellular membrane-delimited organelles as in the case of eukaryotic cells. Therefore, specific proteins, even if in different enzymatic or conformational states, are individual entities occupying distinct locations in the complex bacterial cytoplasm. Single protein studies can probe and exploit this heterogeneity by investigating one molecule at a time. The ability to observe how a single protein behaves inside bacterial cells can help in understanding the action and interaction of proteins in driving larger-scale cellular processes.

Super resolution has been used to determine the spatio-temporal properties of a single bacterial labeled protein [56]. The position of single proteins can be extracted with high precision if their fluorescence emission profiles satisfy the Raleigh criterion (cf. Equation (8)) and they do not change their positions during the acquisition. The fluorescent proteins are localized with a large improvement in resolution (almost an order of magnitude, typically about 30 nm) compared with the resolution of wide-field fluorescence microscopy (250 nm) [56].

Single protein localization is possible when only few proteins are located inside the cell [57]. Acquiring and then localizing the position of the same proteins in serially acquired frames can be used to reconstruct their movements, providing information on their function in vivo [57]. Moreover, if different copies of fluorescent proteins are incorporated into a larger structure, as in a polymeric protein filament, then their super-resolved localization can determine their overall shape and position inside the bacterial cell.

**Figure 6:**
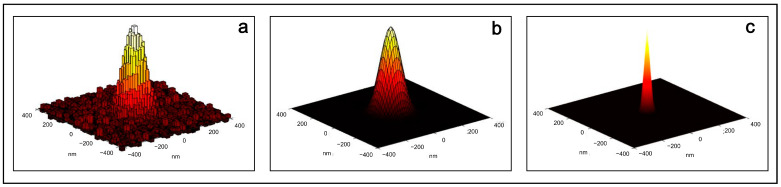
(**a**) The acquired and digitalized PSF of a single yellow fluorescent protein localized in a bacterium; (**b**) its two-dimensional Gaussian fit; and (**c**) the high precision localization of the protein determined from the center of the fitted Gaussian function. Adapted with permission from Ref. [29]. Copyright 2014, Copyright Christoph Braeuchle.

How can a higher precision localization of these proteins be obtained? The position of a single protein is determined by fitting the measured fluorescence intensity profile to a mathematical function. The intensity profile is the diffraction-limited image of a point source of light, and its width is determined by the PSF of the microscope. The measured profile is well approximated by a Gaussian bell-shaped curve, which makes it possible to estimate the position of the point source, indicated by its maximum. The uncertainty in the position parameter (standard deviations of 10–40 nm are typical for a few thousand detected photons) is much smaller than the width of the diffraction-limited PSF (250–300 nm). Figure 6 shows the acquired and digitalized PSF of a single yellow fluorescent protein localized inside a bacterium (a), its two-dimensional Gaussian fit (b) and the high precision localization of the protein determined from the center of the fitted Gaussian function (c) [56].

### 3.5. Experimental Example 2: STED or Confocal Microscopy, Which Is the Best?

**Figure 7:**
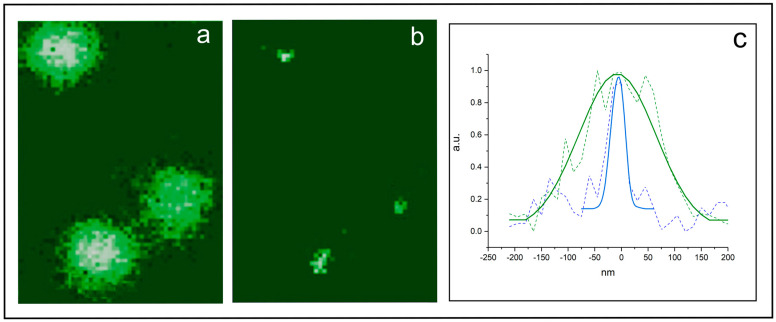
(**a**) Images of synaptic vesicles containing labeled Synaptotagmin proteins acquired by a confocal microscope; (**b**) the same image acquired by a STED microscope, which improves the resolution of single vesicles; and (**c**) the PSF (continuous blue line) of the identified protein patches inside the synaptic vesicles, which are in the range of 25–40 nm, confirming the increase in lateral resolution of the confocal PSF (continuous green line). Adapted with permission from Ref. [29]. Copyright 2014, Copyright Christoph Braeuchle.

Figure 7 shows both the confocal and the STED images of Synaptotagmin I proteins inside synaptic vesicles. The confocal microscope shows diffraction limited spots with a size of about 200 nm (a). The STED image reveals the true size of the spots, which is in the range of 25–40 nm (b). The bottom part of the figure (c) shows the PSF of the identified protein patches inside the synaptic vesicles, which confirms the increase in lateral resolution [58].

## 4. Holotomography

### 4.1. General Description

Holotomography (HTM) is an imaging technique that uses 2D holographic images obtained at various illumination angles to reconstruct a 3D image of a sample at high resolution by means of computer tomography algorithms [59,60,61]. 2D holography is a technique for recording information (phase and amplitude) of an optical wave front (sample beam), which is scattered by a sample and intersects on a detector with a reference beam originating from the same coherent source as the sample beam to create an interference patterns, aka hologram. By illuminating the hologram with the coherent reference beam, the original wave front (i.e., the image of the sample) can be reconstructed.

Holotomography measures the refractive index η of the sample, which provides morphological and biochemical information with sub-micrometric resolution. Since η is an intrinsic optical parameter of every cell, no labeling agents are required. This means that 3D images of a live cell can be recorded over a long time, as long as the physiological conditions are met. Photobleaching and photoxicity do not occur, making HTM very useful for studying the links between structure and function. HTM provides highly reproducible η values in a quantitative manner, and can be utilized for the selection of objects and their visualization. Moreover, η can be directly translated in the quantitative evaluation of cell metrics (e.g., dry weight, volumes, protein concentrations…).

HTM microscopes typically use a low-power continuous-wave laser for illumination and the holograms produced by the sample light scattering are recorded by CCD or CMOS cameras. All the diffracted optical field information (phase and amplitude) contained in the individual hologram are quantitatively retrieved and eventually used to reconstruct the 3D η distribution of the sample from multiple 2D holograms.

HTM has been useful for studying the structures and dynamics of biological samples, such as cells, tissues and proteins samples including red blood cells [62], yeast [63], bacteria [64,65], phytoplankton [66,67] and eukaryotic cells [68,69].

Holotomography should not to be confused with 3D holography. Though 3D holography is a more advanced form of holography, as it allows for the creation of holograms with depth perception and a realistic representation of the object, it can be used only for imaging solid objects, such as sculptures and buildings, and not to detect the internal structures of biological samples.

### 4.2. Theory

HTM consists in sequentially illuminating a sample by means of a plane wave at various incident angles. Each wave is characterized by a transverse incident wave vector k_inc_ and amplitude A_inc_; different far-field diffracted images k are measured for each wave. HTM reconstructs the 3D distribution of the sample refractive index η from the multiple 2D holograms. The reconstruction is obtained by calculating the relative permittivity ε_r_ = η^2^ of the sample and retrieving the set of the diffracted far-field complex amplitude images e(k,k_inc_) according to the following Equation:

e(k,k_inc_) = 8π^3^ A_inc_ (*k*_0_)^2^ Ũ(k − k_inc_) (18)

where *k*_0_ is the wavenumber 2π/λ and Ũ is the Fourier coefficient of the contrast of permittivity U = 1 − ε_r_. In fact, under the Born approximation, the diffraction theory states that there exists a one to one correspondence between the diffracted far-field amplitude image e(k,k_inc_) and the Fourier coefficient of the contrast of sample permittivity_._

The relationship between the opto-geometrical characteristics of a sample and its diffracted field is at the core of most imaging systems that use waves. Their mathematical formulation is very complex and is far beyond the scope of this review; for a detailed description, please refer to Haeberlé et al. (2010) [60]. It is worthwhile stressing that the first zero of the PSF of a holotomograph is positioned at about ≈0.35 λ; this zero determines a lateral resolution of about 0.3 λ/NA, improved (about twice) with respect to the lateral resolution of a classical wide-field microscope (cf. Equation (8)).

### 4.3. A Working Set-Up

Essentially, the optical setup for HTM consists of two parts: the illumination or sample modulation unit and the optical field recording unit. The optical field, which contains both the amplitude and phase information, is recorded employing the principle of interference. Diverse configurations are available for the optical field recording unit, such as off-axis interferometry and phase-shifting interferometry. For systematic control of the angle of the illumination beam, different beam scanning devices can be used, such as the dual-axis galvanometer mirror, the liquid crystal spatial light modulator or the digital micro-mirror device (DMD). The 2D optical field at the sample plane is quantitatively retrieved from the hologram using an algorithm based on a fast Fourier transform method according to Equation (18) [70]; the 3D η distribution map is reconstructed from the retrieved 2D optical field [71,72,73]. A complete description of 2D field retrieval algorithms can be found in [74,75,76].

**Figure 8:**
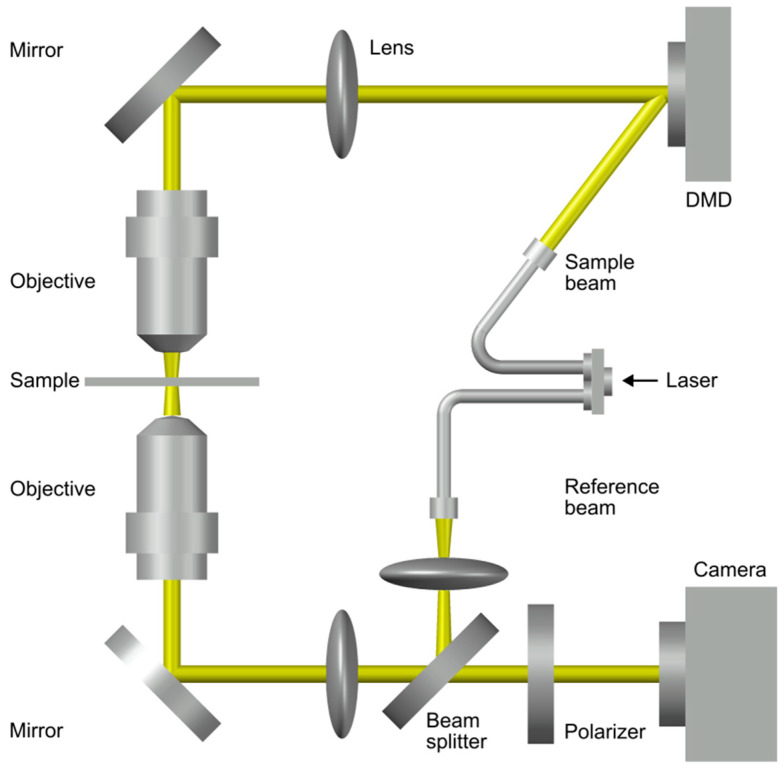
Optical setup of HTM. Adapted with permission from Ref. [67]. Copyright 2018, Copyright Yong Keun Park.

Figure 8 shows an example of an optical system used for HTM. This system uses a diode-pumped solid-state laser (usually a 532 nm laser in vacuum) as a light source. By means of a fiber couple, the laser beam is split into two optical paths, a sample beam and a reference beam. The angle of illumination plane is controlled by a DMD, placed on the sample conjugated plane. The maximum incident angle is 45°. The missing information due to limited illumination and detection angles of the optical lenses is dealt with by an iterative non-negativity constraint [77]. The illumination planes at various incident angles are focalized on the sample via a 1.2 NA condenser lens and collected by a 1.4 NA objective lens. The sample beam and reference beam are then combined by a beam splitter, generating a spatially modulated hologram that is focalized onto the sensor plane of a CMOS camera, and then digitalized. Two hundred and one holograms are acquired at a 100 Hz rate. This system has a 150 nm lateral resolution, 350 nm axial resolution and 50 μm × 50 μm maximum field of view, with a total magnification of 60× [78].

### 4.4. Experimental Example 1: Quantitative Measurement of Lipid Contents in Individual Microalgal Cells

HTM is very useful for the quantitative measurement of living cell compartments that exist as condensed phases of biopolymers. HTM allows segmentation (i.e., identification and separation) of these subcellular structures and the 3D reconstruction of the corresponding η distribution.

**Figure 9:**
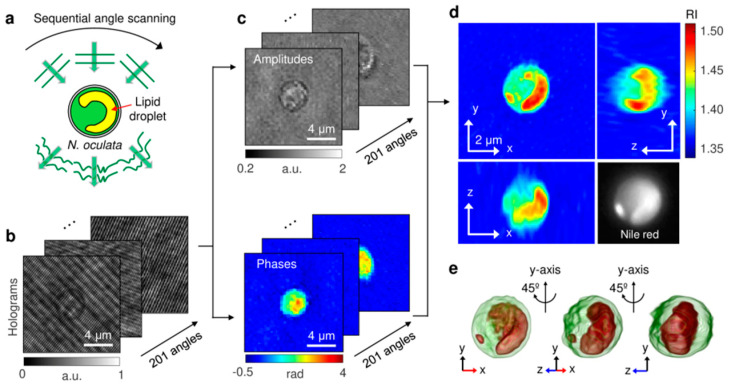
Schematic diagrams of the label-free identification of lipid droplets in individual *N. oculata* cells using HTM: (**a**) The sample is consecutively illuminated by a plane wave at various incident angles. (**b**) The holograms are recorded at 201 incident angles. (**c**) Retrieved amplitudes and phases of the optical fields diffracted by the sample. (**d**) Maps of the reconstructed 3D η distribution of *N. oculata* in the x-y, y-z and x-z planes. The Nile red fluorescence image of the same cell is shown in the lower right corner for comparison. (**e**) The 3D rendered iso-surface image of the reconstructed η distribution at various viewing angles. Adapted with permission from Ref. [67]. Copyright 2018, Copyright Yong Keun Park.

Park et al. [67] identified and quantified the lipid content of the living microalga *Nannochloropsis oculata*, using 3D η distribution maps. They also investigated the morphological and composition alteration of the alga under nitrogen deficiency conditions (NDF), which trigger endocellular lipid accumulation [79,80] (Figure 9). The high η regions (η > 1.46) were identified as lipid droplets, on the basis of the comparison with the fluorescence images of the same microalga labeled with Nile red [67]. This high η value corresponded to the average η of vegetable oils [81]. To quantitatively evaluate the lipid accumulation process and the effects of NDF, the authors retrieved cell volume, dry cell weight, lipid weight and lipid ratio of individual cells from the measured 3D η distributions [67]. The lipid weight inside the cell was obtained by multiplying the volume of the identified lipid regions by the average density of vegetable oils (0.9 g/mL). The weight of the non-lipid components was calculated using a parameter called refractive index increment (η_i_), which is a constant that indicates the variation of η with the solute concentration [82]. The refractive index of non-lipid regions could be expressed and calculated using Equation (19):

η(x, y, z) = η_water_ + Σ_i_ α_j_C_j_(x, y, z) (19)

where η_water_ represents the η of water and α_j_ and C_j_ indicate the η_i_ and concentration of an arbitrary component j, respectively. Using the average η_i_ of 0.185 mL/g, which is a typical value for proteins and is comparable to that of nucleic acid, sugars and salts, the total concentration of the non-lipid components C_j_(x, y, z) could be obtained [67]. The weight of the non-lipid components could be calculated by integrating the concentration over the non-lipid volume.

The cell volumes were measured to investigate the change in cell sizes. The results showed that the volumes of both the control and NDF cells did not significantly change during cultivation [67]. The measured volumes of *N. oculata* were mostly distributed within the range 10–20 × 10^−9^ μL; the smallest and largest cells had volumes of approximately 5 and 33 × 10^−9^ μL, which correspond to an equivalent diameter of 2.2 and 4.0 μm, respectively. These figures were consistent with the cell diameters measured using a Coulter counter [67].

The dry cell weights of the cells were measured and analyzed to evaluate the changes in the weights of the components during cultivation under NDF condition. After three days of NDF cultivation, the average lipid weight inside the cells increased 2.5 times. This result is consistent with the fact that lipid accumulation is induced by nitrogen starvation [83,84]. To validate the values retrieved from HTM, the lipid weight in the bulk volume was also measured using gas chromatography and converted to the lipid weight per cell using the cell number [67].

### 4.5. Experimental Example 2: The 3D Structure of Euglena Gracilis Photoreceptor

The life of photosynthetic microorganisms as algae depends on energy from the sun, which is also a source of information for orienting them in space and guiding their movement and growth. Hence, light becomes a sensory stimulus governing the behavior of algae in their environment towards areas matching their irradiation requirements. To perceive, store and exploit the information light contains, proper devices are needed, the photoreceptors, which perform perception, transduction, amplification and transmission. Thanks to photoreceptors, algae can “see”, i.e., they can process the light stimulus reaching them and transform it into an oriented movement. Though these devices do not possess the optical requirements necessary to form images as in higher organisms, still they share functional similarities with these vision systems. They do possess a photoreceptive part, which in most unicellular algae consists of 2D patches of photoreceptive protein; a screening part, consisting of orange–red pigments organized in granular form; and a transduction part, which transmits the light signal to the effector, eventually controlling the cell movement. Only in few groups, the photoreceptor is detectable by light microscopy as a distinct organelle in a range size of 1–2 μm [85,86].

**Figure 10:**
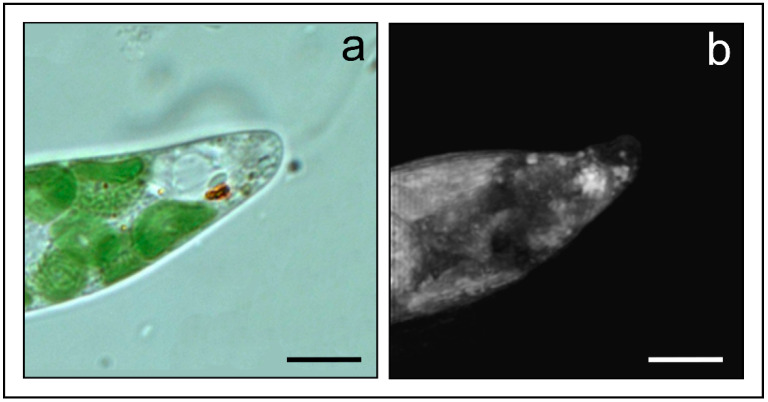
(**a**) Apical region of *Euglena gracilis* in WFM. The orange spot localizes the eyespot, but the granules cannot be identified; (**b**) the same region of another cell acquired by HTM: the eyespot granules are visible as separate entities. Scale bar: 10 μm.

The iconic example of an algal photoreceptor is that present in the unicellular alga *Euglena gracilis* (Euglenophyceae). This alga possesses a 1 μm^3^ lens-shaped photoreceptor hidden and protected inside the apical portion of the cell by the eyespot, which is a screening device consisting of carotenoid containing granules. Holotomograpy is the ideal method of investigation for this structure because it can circumvent the difficulties due to the photoreceptor position, thus allowing imaging of the organelle in vivo and in its native state without staining or labeling. The higher resolution capability of this technique with respect to WFM is clarified by Figure 10. HTM guarantees the proper visualization of structural details (single eyespot granules), helping the correct physiological interpretation of the photoreception and transduction mechanisms.

**Figure 11:**
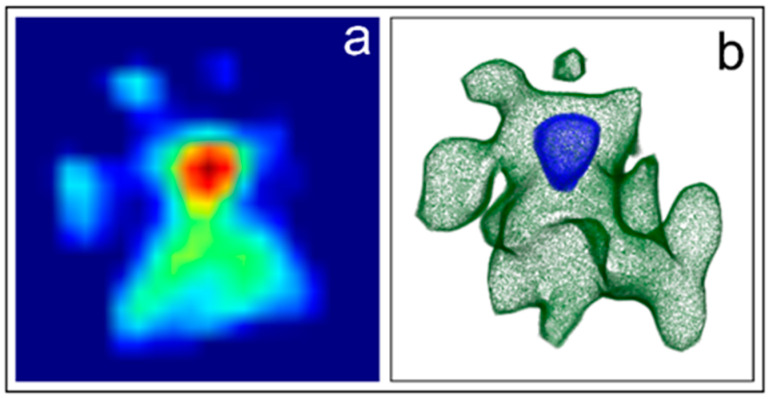
(**a**) a single hologram produced by HTM: the green spots surround the bright red of the photoreceptor; (**b**) 3D reconstruction of the η distribution map of the whole photoreceptive apparatus of *Euglena*, showing the photoreceptor (blue) nested and surrounded by the eyespot (green). Adapted with permission from Ref. [19]. Copyright 2023, Copyright Paolo Gualtieri.

The 3D reconstruction of the photoreceptive apparatus of *Euglena* supports the role of the eyespot as a selective screening device of the wavelengths impinging on it and reaching the photoreceptor [13] (Figure 11). This reconstruction also suggests a possible localization of the photoreceptor in a focal position with respect to the hemi-elliptic disposition of the granules, in such a way that the light reaching the photoreceptor from a direction opposite to the eyespot can be reflected back onto the organelle by the pigmented granules, thus increasing the intensity of the light impinging on it. HTM calculated a refractive index value of 1.46 for the eyespot granules, consisting of lipid droplets, and a refractive index value of 1.50 for the photoreceptor, which correspond to a weight of about 0.25 pg. This weight is in good agreement with the weight of 5 × 10^6^ photoreceptive proteins of 30 kD calculated by microspectrophotometric measurements and protein isolation and purification procedures [17,87].

## 5. Conclusions

In this review, we have presented three different quantitative microscopy set-ups that allow measuring and calculating quantitative parameters in vivo such as the absorption spectra of subcellular compartments (experimental examples Section 2.4 and Section 2.5); the high precision localization of fluorescent proteins inside vesicles (experimental examples Section 3.4 and Section 3.5); the evaluation of subcellular volume and weight, cell volume, dry cell weight and cellular component weight from measured 3D η distributions (experimental examples Section 4.4 and Section 4.5). These quantitative microscopy tools are a powerful approach to improve non-destructive investigations of cellular and subcellular properties (both physical and chemical) at macromolecular scale resolutions.

## Figures and Tables

**Table 1:** Overview of the major quantitative SRLM techniques and their characteristics.

	Lateral Resolution (nm)	Axial Resolution (nm)	Methods of Illumination	Methods of Image Generation	Fluorescent Probe	Five Aspects of Microscopy	Merits	Limitations
WFM	250	700	Lamp	Direct visualization	Conventional	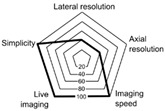	Perfect for living cell Very easy to use	Low axial and lateral resolution
CLSM	180–250	500	Pinhole	Scanning excitation beam	Conventional	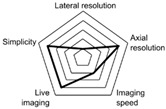	High axial resolution 3D imaging capability Reduced photobleaching	Limited field of view Slow processing
2D-STED	40–60	600	Hardware shaped excitation beam	Scanning excitation beam	Photo switchable	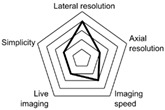	Resolution directly improved with PSF to ~50 nm 2 colour imaging (more if combined with other advanced techniques) No post-processing Lateral resolution 20–70 nm 20 micron depth penetration	Photobleaching, Phototoxic Requires very stable dyes Difficult for live cells Requires complex alignment Low dynamic range
SIM	100–125	2D 500–700 3D 250–350	Patterned wide field	Multiple images combining Fourier space	Conventional	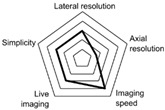	Live cell imaging Multicolour imaging No special sample preparation Simple to use Fast	Require sensitive cameras Photobleaching/toxicity Subject to artifacts Limited sample thickness
PALM/STORM	10–50	500–700	Stochastic fluorophore excitation	Multiple single molecules frames	Photoactivable	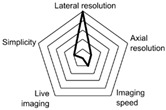	Single molecule sensitivity Highest potential resolution Multichannel imaging Low illumination power	Specific fluorophores required Shallow depth-limited Long acquisition times Impractical for live cell

## Data Availability

Not applicable.
